# Therapeutic recommendations in *HFE* hemochromatosis for p.Cys282Tyr (C282Y/C282Y) homozygous genotype

**DOI:** 10.1007/s12072-018-9855-0

**Published:** 2018-03-27

**Authors:** Paul Adams, Albert Altes, Pierre Brissot, Barbara Butzeck, Ioav Cabantchik, Rodolfo Cançado, Sonia Distante, Patricia Evans, Robert Evans, Tomas Ganz, Domenico Girelli, Rolf Hultcrantz, Gordon McLaren, Ben Marris, Nils Milman, Elizabeta Nemeth, Peter Nielsen, Brigitte Pineau, Alberto Piperno, Graça Porto, Dianne Prince, John Ryan, Mayka Sanchez, Paulo Santos, Dorine Swinkels, Emerência Teixeira, Ketil Toska, Annick Vanclooster, Desley White, Contributors and Hemochromatosis International Taskforce

**Affiliations:** 1 University Hospital, Western University, London, ON Canada; 2 Asociación Española de Hemocromatosis, Santa Coloma, Spain; 3 Inserm Unit 1241, University Hospital Pontchaillou, Rennes, France; 4 HI - Haemochromatosis International, London, UK; 5 European Federation of Associations of Patients with Haemochromatosis, Seine, France; 6 Haemochromatose-Vereinigung Deutschland, Hürth, Germany; 7 International Bioiron Society, Schaumburg, USA; 8 Hebrew University of Jerusalem, Jerusalem, Israel; 9 Division of Hematology, Santa Casa Medical School of Sao Paulo, Sao Paulo, SP Brazil; 10 Department of Medical Biochemistry, Oslo University Hospital, Oslo, Norway; 11 Department of Electronic and Computer Engineering, School of Engineering and Design, Brunel University, Uxbridge, UK; 12 The Haemochromatosis Society, Hertfordshire, UK; 13 David Geffen School of Medicine, UCLA, Los Angeles, CA USA; 14 Department of Medicine, University of Verona Veneto Region Referral Center for Iron Metabolism Disorders, Verona, Italy; 15 Department of Medicine, Unit of Gastroenterology and Rheumatology, Karolinska Institutet, Stockholm, Sweden; 16 Division of Hematology/Oncology, University of California, Irvine, CA USA; 17 Haemochromatosis Australia, Meridan Plains, Australia; 18 Danish Haemochromatosis Association, Copenhagen, Denmark; 19 Center for Iron Disorders, David Geffen School of Medicine, UCLA, Los Angeles, CA USA; 20 Institut für Biochemie und Molekulare Zellbiologie, University Medical Center Hamburg, Hamburg, Germany; 21 Fédération Française des Associations de Malades de l’hémochromatose, Paris, France; 22 Department of Medicine and Surgery, Centre for Rare Diseases, University of Milano-Bicocca, Monza, Italy; 23 Association for the Study of Hemochromatosis and Iron Overload Diseases, Monza, Italy; 24 IBMC – Instituto de Biologia Molecular e Celular, Universidade do Porto, Porto, Portugal; 25 Oxford University Hospitals NHS Foundation Trust, John Radcliffe Hospital, Oxford, UK; 26 Department of Pharmacology, Universidade Federal de Sao Paulo, São Paulo, Brazil; 27 Laboratory of Genetics and Molecular Cardiology, Heart Institute (InCor), University of São Paulo Medical School, 03 de Maio St. INFAR, 4° andar - Vila Clementino, São Paulo, SP Brazil; 28 Department of Laboratory Medicine, Radboudumc, Nijmegen, The Netherlands; 29 i3S - Instituto de Investigação e Inovação em Saúde, Universidade do Porto, Porto, Portugal; 30 Norwegian Haemochromatosis Association, Bergen, Norway; 31 University Hospitals Leuven, Gasthuisberg, Louvain, Belgium; 32 University of Plymouth, Plymouth, UK; 33 CHP-HSA - Centro Hospitalar do Porto - Hospital Santo António, Porto, Portugal; 34 FCUP - Faculdade de Ciências da Universidade do Porto, Porto, Portugal; 35 APH - Associação Portuguesa de Hemocromatose, Porto, Portugal; 36 Haemochromatose Vereniging Vlaanderen, Leuven, Belgium

## Abstract

Although guidelines are available for hereditary hemochromatosis, a high percentage of the recommendations within them are not shared between the different guidelines. Our main aim is to provide an objective, simple, brief, and practical set of recommendations about therapeutic aspects of *HFE* hemochromatosis for p.Cys282Tyr (C282Y/C282Y) homozygous genotype, based on the published scientific studies and guidelines, in a form that is reasonably comprehensible to patients and people without medical training. This final version was approved at the Hemochromatosis International meeting on 12th May 2017 in Los Angeles.

## Introduction

Although guidelines are available for hereditary hemochromatosis (HH), a high percentage of the recommendations within them are not shared between the different guidelines [1]. Our main aim is to provide an objective, simple, brief, and practical set of recommendations about therapeutic aspects of *HFE* hemochromatosis for p.Cys282Tyr (C282Y/C282Y) homozygous genotype, based on the published scientific studies and guidelines, in a form that is reasonably comprehensible to patients and people without medical training.

The final version of these recommendations was approved at the Hemochromatosis International meeting on 12th May 2017 in Los Angeles.

## Whom to treat and when to start

Patients with *HFE* p.Cys282Tyr (C282Y/C282Y) homozygous genotype and biochemical evidence of iron overload, i.e., increased serum ferritin (> 300 µg/L in male and postmenopausal female and > 200 µg/L in premenopausal female) and increased fasting transferrin saturation (≥ 45%) [2, 3].

### Considerations

- A judgement has to be made for each individual patient taking into account their ferritin level (according to local reference value), age, gender, and co-morbidities.
- Patients with other genotypes should be referred for further advice.
- Recent studies observed a beneficial effect of early and sustained management of patients with iron excess, even when iron load is mild or moderately elevated serum ferritin [4, 5].
- Elevated serum ferritin values are very common and the most frequent causes are not associated with HH, but with metabolic syndrome inflammation (ferritin is an acute-phase protein), alcoholism, and liver damage. Thus, it is critical to investigate rigorously the cause of high serum ferritin values. Searching for increased plasma transferrin saturation is critical for the diagnosis of HH, since it corresponds to the basic and earliest biochemical abnormality in this disease.
- Magnetic resonance imaging (MRI), when available, may be used to quantify iron overload in the liver (or in other organs).

## Treatment

Phlebotomy (venesection therapy) is the standard treatment for patients with HH having been used for more than 60 years. It is effective in reducing morbidity and mortality of HH [6].

Iron overload leads to tissue injury mainly through the production of reactive oxygen species which damage cell membranes and organelles, ending up with cellular death. Thus, to start the treatment is important.

Each 500 mL phlebotomy withdraws approximately 250 mg of iron which is subsequently released, in a compensatory process, from overloaded tissues (especially the liver), ending up, with phlebotomy repetition, to the total removal of body iron excess.

## How to treat

### Initial or induction phase

A phlebotomy schedule of the order of 400–500 mL, considering body weight, weekly or every 2 weeks has been proposed [2, 7].

The objective in this phase is usually to reach serum ferritin at 50 µg/L provided that there is no anemia.

Serum ferritin should be checked once a month until the values reach the upper normal limits, and every 2 weeks thereafter, until the final goal of serum ferritin is reached [2, 3, 5].

#### Considerations

- The volume and frequency of the phlebotomies should be adapted to the clinical characteristics and (individual) tolerance of the patient.
- Tolerance: clinical data (general tolerance and blood pressure); hemoglobin (levels should not decrease below 11 g/dL) [7].
- Phlebotomies can be performed in a clinic, hospital, transfusion/blood donor centers or in certain circumstances at home (by a nurse under medical supervision).
- Patients should be well hydrated and fed.

### Maintenance phase

The maintenance phase follows the induction phase.

The patient with HH needs lifelong follow-up.

One phlebotomy every 1–4 months, depending on the patient’s iron status [2, 7].

Efficacy: the usual aim is to maintain ferritin levels around 50 µg/L (without anemia) [2, 3, 5].

#### Considerations

- The frequency of maintenance phlebotomy varies among individuals, ranging from one per month to one per year [3].
- Hemoglobin levels should not be < 11 g/dL.
- Hemoglobin value, typically, should be assessed preceding phlebotomy, especially in older patients, who are more susceptible to anemia and chronic blood losses.
- Serum ferritin should be checked at every other phlebotomy (at the same time as hemoglobin), at least yearly.
- Fasting transferrin saturation should also be checked, at least once a year.
- In certain countries, a growing number of patients with uncomplicated hemochromatosis donate blood or qualify as blood donors. Blood removed from therapeutic phlebotomy may be used at the discretion of the blood service [8].

## When to stop

Patients who have had iron overload should never stop having their iron status monitored and their treatment planned in the light of their iron status, general condition, and age.

## Additional considerations

### Diet

A healthy varied diet should be eaten, avoiding foods with iron fortification such as breakfast cereals. Iron and vitamin C supplementation and high alcohol consumption should also be avoided [3]. In certain geographical regions (especially subtropical), HH patients should avoid raw shellfish and undercooked pork because of the risk of severe infections.

Dietary restriction should not replace phlebotomy therapy.

### Chelation therapy

Iron chelation is usually indicated for iron overload related to chronic anemias that need repeated transfusions. However, iron chelators are an alternative treatment (or adjuvant) only used in rare and special cases of HH, when phlebotomies are medically contraindicated, simply impossible owing to poor vein conditions, or the efficacy was not achieved with phlebotomies.

## Possible future of HH therapeutics

Hepcidin-based therapies might, in future, become a potential adjunct treatment to phlebotomy in the induction phase or a replacement for the phlebotomy maintenance phase. The possibility of using hepcidin is based on the diminishing of hepcidin levels in HH patients, which is responsible for increased serum iron and transferrin saturation, and subsequently to tissue iron overload [9].
